# Genetic codes optimized as a traveling salesman problem

**DOI:** 10.1371/journal.pone.0224552

**Published:** 2019-10-28

**Authors:** Oliver Attie, Brian Sulkow, Chong Di, Weigang Qiu

**Affiliations:** 1 Department of Biological Sciences, Hunter College, City University of New York, New York, United States of America; 2 Graduate Center, City University of New York, New York, United States of America; 3 Department of Physiology and Biophysics & Institute for Computational Biomedicine, Weil Cornell Medical College, New York, New York, United States of America; University of Leeds, UNITED KINGDOM

## Abstract

The Standard Genetic Code (SGC) is robust to mutational errors such that frequently occurring mutations minimally alter the physio-chemistry of amino acids. The apparent correlation between the evolutionary distances among codons and the physio-chemical distances among their cognate amino acids suggests an early co-diversification between the codons and amino acids. Here we formulated the co-minimization of evolutionary distances between codons and physio-chemical distances between amino acids as a Traveling Salesman Problem (TSP) and solved it with a Hopfield neural network. In this unsupervised learning algorithm, macromolecules (e.g., tRNAs and aminoacyl-tRNA synthetases) associating codons with amino acids were considered biological analogs of Hopfield neurons associating “tour cities” with “tour positions”. The Hopfield network efficiently yielded an abundance of genetic codes that were more error-minimizing than SGC and could thus be used to design artificial genetic codes. We further argue that as a self-optimization algorithm, the Hopfield neural network provides a model of origin of SGC and other adaptive molecular systems through evolutionary learning.

## Introduction

### Limits of natural selection

Discoveries by Alfred Wallace and Charles Darwin in the 19th century established natural selection as the primary mechanism of evolutionary adaptation [1,2]. The Modern Synthesis and the more recent Neutral Theory of molecular evolution reaffirm the supremacy of natural selection as the only evolutionary mechanism capable of deterministic, directional changes against a backdrop of stochastic, directionless evolutionary forces including mutation, genetic drift, and recombination [3–5]. Yet theories of natural selection and population genetics offer limited explanations on the apparent evolutionary trend towards increasing organizational complexity of living systems, as evident [6] in the repeated emergence of complex and robust structures and molecular processes including molecular pathways, subcellular structures, multicellularity, sexual reproduction, embryonic development, sociality, and self-regulating ecosystems [7,8]. A major weakness of Darwinian natural selection and population genetic analysis is its inability to specify testable algorithmic steps, to replicate with simulation, or to predict the future outcomes of organic complexity [9,10].

In recent years, computational and algorithmic learning emerged as a major contender to bridge the epistemological gap between the feasibility of organismal complexity foretold by the theory of natural selection and its algorithmic plausibility [9–12]. Algorithmic learning, defined as the process of creating internal representations (e.g., as memories or genomes) of external regularities through “concrete computation that takes a limited number of steps”, applies equally well to understand the biological origins of cognition and adaptation [10]. For example, the Multiplicative Weights Update learning algorithm has been shown to be equivalent to natural selection on genotypes in asexual populations and on individual alleles in sexual populations [13,14]. By quantifying the degree of computational complexity of the problems imposed by environments, computational learning has not only the potential to generate complex adaptations but also to predict the limit and adverse consequences of adaptations [10].

### Evolutionary connectionism

Computational learning is at the heart of a new evolutionary paradigm termed “evolutionary connectionism”, which posits a theoretical equivalence between learning algorithms and evolutionary processes leading to the emergence of complex adaptations [11,15]. Central to evolutionary connectionism is the concept of correlational or associative learning first proposed by Donald Hebb in understanding the spontaneous origin of neural networks capable for memory [16]. The Hebbian rule, known colloquially as “cells that fire together, wire together”, is now postulated as a general pattern-generating mechanism in living systems beyond neural systems. For example, the origin of developmental pathways and other gene regulatory networks may be a consequence of following genomic analogs of the Hebbian rule that “genes that are selected together wire together” and “genes that function together junction together” [17]. Bacterial operons, consisting of tandemly arranged and co-transcribed genes, may be a physical manifestation of the Hebbian rule in shaping genome structure (“genes that function together locate together”).

Despite (or perhaps because of) its simplicity, the Hebbian correlational learning has considerable power in generating adaptive and robust features in living systems beyond neural networks. First, as an unsupervised learning algorithm, Hebbian learning is efficient in finding locally optimal solutions without the need to search through a prohibitively large number of possible solutions and test these solutions individually, as is necessary in the process of natural selection. Second, as a distributed algorithm, Hebbian learning occurs locally between directly interacting entities (neurons or genes) while nonetheless leading to emergence of system-level optimal structures (neural or gene networks). It is not necessary to hypothesize that the system-level phenotypes (e.g., developmental processes or gene networks) are themselves direct targets of natural selection. Third, by discovering and encoding trait correlations, Hebbian learning results in networks with foresightedness and robustness [11].

For the time being, however, evolutionary connectionism has primarily been a conceptual framework. It remains unclear how extensively Hebbian learning–relative to natural selection and other learning algorithms–operates in shaping molecular and cellular structures beyond neural systems. It is desirable and indeed necessary to test claims of evolutionary learning in the context of a specific molecular system with computational, statistical and biological evidence.

### Origin of genetic code

**Fig 1:**
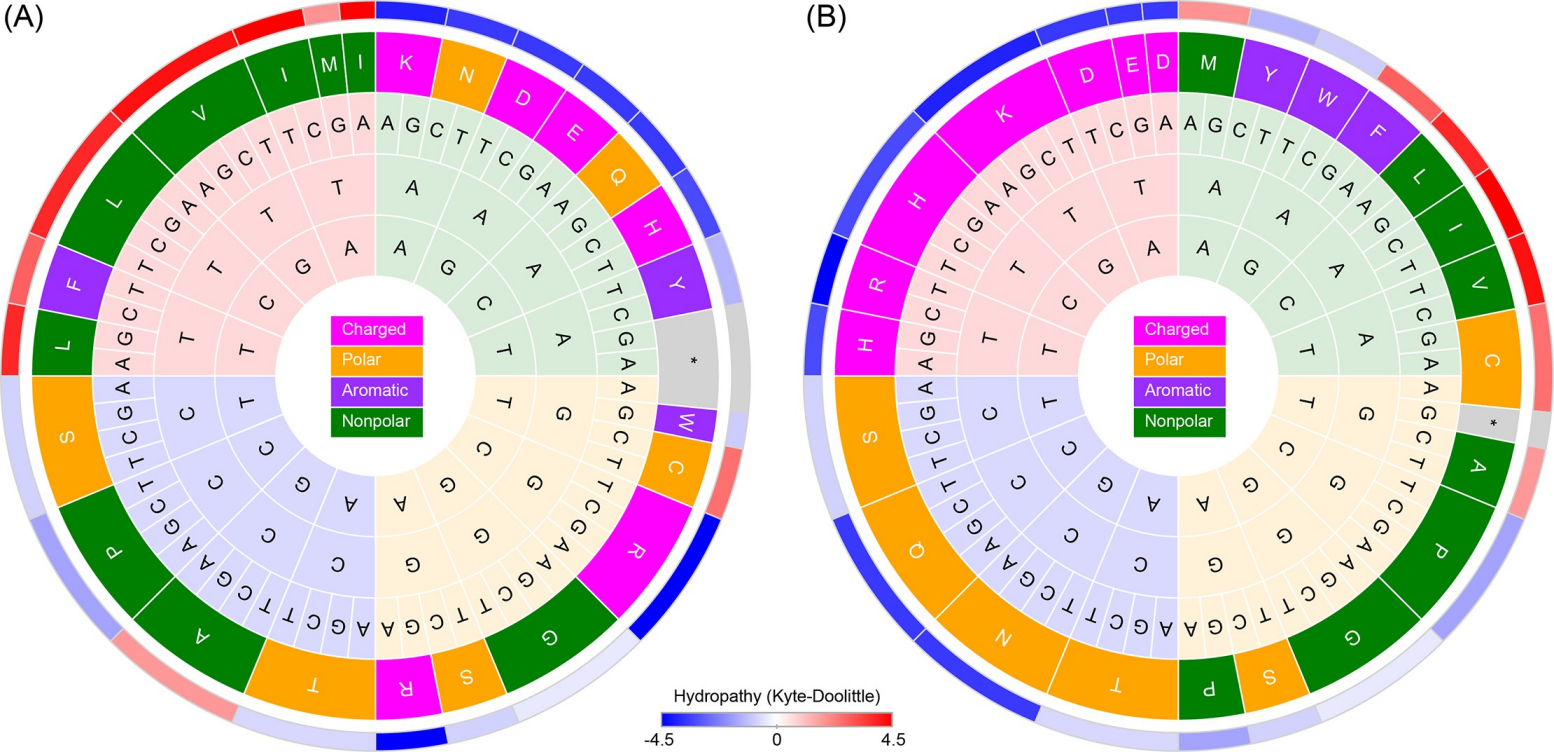
**Codon wheels showing (A) the standard genetic code (SGC) and (B) a Hopfield-optimized genetic code.** Bases are arranged (from center outward) in the order of 1^st^, 2^nd^, and 3^rd^ codon positions. Within each of these three rings, the four bases are cycled clockwise in the order of “AGCT” to minimize the number of transversions. The codon wheel represents the shortest mutation path and is modeled as the time dimension in the Traveling Salesman Problem (TSP) representation of the genetic codes. In the 4^th^ ring, amino acids are colored according to a physio-chemical classification [18]. In the 5^th^ ring, amino acids are colored by the Kyte-Doolittle measure of hydrophobicity [19].

Here we test the hypothesis that the standard genetic code may have been evolved predominantly through a self-learning rather than a natural-selective process. The standard genetic code (SGC, Fig 1A), with which 64 possible combinations of triple nucleotides (“codons”) encode 20 canonical amino acids and the translational stop signal, underlines much of the robustness of organisms against deleterious mutations. For example, single-nucleotide mutations occurring at the 3^rd^ codon position typically result in no change of amino acids. Such properties of SGC are called “error-minimization”, referring to the fact that SGC is non-randomly structured to minimize disruptions by DNA mutations to protein sequences, structures, and functions.

Although there is a consensus on the adaptiveness of SGC [20–25], the molecular processes that led to its origin remain controversial [20,22,24,24–31]. Natural selection, in conjunction with chemical processes during early history of life, has been the most frequently argued hypothesis to explain the origin of the highly optimized and nearly universal SGC [26,30,32]. However, the selective mechanism tends to be slow and inefficient because it implies that SGC emerges from competition among cells equipped with random genetic codes [33]. More contentiously, because the protein translation machinery is a complex subcellular system consisting of, among others, tRNAs, aminoacyl-tRNA synthetases (aaRSs) and the ribosome, the selective hypothesis raised the question whether (and how) natural selection operates at the level of individual genes, the subcellular system, or the cell system as a whole. For example, it has been argued that the apparent adaptiveness of SGC may have risen as a by-product of incremental evolution through codon capture, during which structurally similar aaRSs tend to recognize phylogenetically similar tRNAs and physio-chemically similar amino acids [6,12,34–36]. Regardless, neither the selective hypothesis nor the incremental evolution hypothesis directly addresses the algorithmic processes by which error-minimizing capacity of SGC may have evolved. Besides being of considerable evolutionary interest, the design principle and algorithmic origin of SGC are of practical importance for reengineering the genetic code to create synthetic therapeutic proteins using non-canonical amino acids [37,38].

Here, we explore the possibility of an evolutionary origin of SGC through self-optimization. The search for optimal genetic codes by using learning algorithms is not new and studies have concluded that SGC is far from being globally or even locally optimal [20,24–26,31,39–41]. Our specific objective is to test if evolutionary connectionism (and the Hebbian learning rule in specific) could lead to error-minimizing genetic codes.

## Models & methods

### Hopfield network & traveling salesman problem

Hopfield neural network is an algorithmic model of associative memory that implements the Hebbian learning rule [42]. A Hopfield neural network, consisting of symmetrically connected neurons, is capable of storing, retrieving, and restoring memories when activities of individual neurons are determined by the Hebbian learning rule [42,43]. For example, in a binary Hopfield network where each neuron’s activity takes the value of either 1 or -1, the connection weight (*w*_*i*,*j*_) between a pair of neurons is increased if their activities (*x*_*i*_ and *x*_*j*_) are positively correlated (both 1 or both -1) and decreased if negatively correlated (one 1 and the other -1). Once connection weights are specified, a random initial state the network would evolve towards a locally stable state where the following definition of the network energy is at the minimum [43]:

$$E=-\frac{1}{2}\sum_{i,j}w_{i,j}x_{i}x_{j}$$ (1)

As such, while the neuron activities are determined at local levels through interactions with each other, the neural network as a system displays collective, emergent behavior mimicking associative memory. The network is able to, for example, retrieve a complete state from incomplete inputs or recover a correct state from inputs with errors. The system is also robust in the sense that the network functions well even when some of the neurons are removed, mimicking a damaged brain and indicating encoded information redundancy [43].

Besides being a powerful model of associative memory, Hopfield network is an efficient algorithm for solving combinatorial optimization problems, such as the traveling salesman problem (TSP), which is to find the shortest tours to cover *N* cities while visiting each city once [44]. Instead of being a memory device, here the Hopfield network was used as a computational tool to search for combinatorial optimal states. To solve the TSP, for example, a Hopfield network is simulated with *N*^*2*^ neurons, each representing the probability of a city being visited at a tour position. An energy function is defined to reflect both the constraints (e.g., each city to be visited once and the salesman can visit only one city at a time) and a measurement to be minimized (e.g., the tour length). When initialized with arbitrary activities, the network progressively reaches a minimum representing a locally shortest path as the energy function converges to a stable local minimum [44,45].

The original Hopfield-Tank algorithm was found difficult to replicate as the number of cities increased and a more efficient algorithm using neural normalization and simulated annealing was proposed [46]. The mean field of a neuron representing city *X* visited at tour position *i* is defined as:

$$E_{Xi}=d_{p}\sum_{Y\neq X}v_{Y_{i}}+\sum_{Y\neq X}d_{XY}{(v}_{Y,i+1}+v_{Y,i-1})$$ (2),

where *d*_*p*_ is a penalty experimentally adjusted to ensure that only one city can occupy a tour position and v_X*i*_ is the probability of city *X* at tour position *i*. Values of *v*_*xi*_’s are assumed to obey a Boltzman distribution at any given simulated temperature *T*: $v_{Xi}\propto e^{-E_{Xi}/T}$. At each iterative updating step, the neuron outputs are normalized to sum up to one so that each value represents a true probability:

$$v_{Xi}=\frac{e^{-E_{Xi}/T}}{\sum_{j}e^{-E_{Xj}/T}}$$ (3)

Finding a valid path depends on the temperature *T* and the penalty *d*_*p*_. As the temperature increases, neuron outputs become increasingly uniform (*v*_*Xi*_→1/*n*). As the temperature drops to a critical value (*T*_*0*_), the neurons anneal to a steady low-energy state representing a stable, locally minimal-energy mapping between the cities and tour positions. Both the critical temperature *T*_*0*_ and *d*_*p*_ that lead to valid tours were experimentally determined by simulations as in all other Hopfield solutions of the TSP [46]. The initial values of *v*_*Xi*_ influence whether the network converges. To ensure convergence, we used initial voltage values of 0.5 plus a random number in between -0.1 and +0.1 and *d*_*p*_ = 0.7.

### Algorithm to generate optimal genetic codes using TSP

#### TSP model of SGC origin

We modeled a genetic code as a tour in the Traveling Salesman Problem (TSP) so that it could be optimized by using a Hopfield neural network. We hypothesized that the optimal genetic code minimized the total distance of a tour of 20 amino acids from one codon to next, analogous to a tour of *N* cities by a salesman from one time point to the next. To make the codons equivalent to the linearly ordered time points in a TSP, it was necessary to generate a linearly ordered sequence of codons that minimizes the cumulative mutational distance between codons (see “codon wheel” below).

To represent genetic codes as a TSP, we first constructed a Hopfield network consisting of 21 x 21 neurons, the activity of each of which representing the probability of an amino acid (“cities”) at a tour position. We measured distances between two amino acids (*X* and *Y*) as the Euclidean distance: $d_{XY}=\sqrt{\sum_{i}{\left(X_{i}-Y_{i}\right)}^{2}}$, where *i* stands for one of the physio-chemical indices (either hydrophobicity, polarity, volume, or isoelectric point; Table 1). To remove effects of difference in magnitudes when combining multiple indices, we rescaled each index by normalizing the values to a mean of zero while maintaining the standard deviation. Values for stop codons were arbitrarily assigned to be an outlier (greater or less than two standard deviations from the mean).

**Table 1:** Amino acid indices.

1-letter code	3-letter code	Polarity^*a*^	Hydrophobicity^*a*^	Volume^*a*^	Iso-electric point^*a*^
A	Ala	7.0 (-0.1672)	1.8 (0.7638)	31 (-1.067)	6.00 (-0.02972)
C	Cys	4.8 (-1.043)	2.5 (1.002)	55 (-0.5477)	5.07 (-0.5561)
D	Asp	13.0 (2.221)	-3.5 (-1.039)	54 (-0.5694)	2.77 (-1.858)
E	Glu	12.5 (2.022)	-3.5 (-1.039)	8.3 (-1.559)	3.22 (-1.603)
F	Phe	5.0 (-0.9631)	2.8 (1.104)	132 (1.120)	5.48 (-0.3241)
G	Gly	7.9 (0.1910)	-0.4 (0.01531)	3 (-1.674)	5.97 (-0.0467)
H	His	8.4 (0.3900)	-3.2 (-0.9373)	96 (0.3402)	7.59 (0.8703)
I	Ile	4.9 (-1.003)	4.5 (1.682)	111 (0.6651)	6.02 (-0.0184)
K	Lys	10.1 (1.067)	-3.0 (-0.8693)	119 (0.8384)	9.74 (2.087)
L	Leu	4.9 (-1.027)	3.8 (1.444)	111 (0.6651)	5.98 (-0.04104)
M	Met	5.3 (-0.8437)	1.9 (0.7978)	105 (0.5352)	5.74 (-0.1769)
N	Asn	10.0 (1.027)	-3.5 (-1.039)	56 (-0.5261)	5.41 (-0.3637)
P	Pro	6.6 (-0.3264)	-1.6 (-0.3930)	32.5 (-1.035)	6.30 (0.1401)
Q	Gln	8.6 (0.4696)	-3.5 (-1.039)	85 (0.102)	5.65 (-0.2278)
R	Arg	9.1 (0.6686)	-4.5 (-1.380)	124 (0.9466)	10.76 (2.665)
S	Ser	7.5 (0.03184)	-0.8 (-0.1208)	32 (-1.046)	5.68 (-0.2108)
T	Thr	6.6 (-0.3264)	-0.7 (-0.08676)	61 (-0.4178)	6.16 (0.06085)
V	Val	5.6 (-0.7243)	4.2 (1.580)	84 (0.08035)	5.96 (-0.05236)
W	Trp	5.2 (-0.8835)	-0.9 (0.1548)	170 (1.943)	5.89 (-0.09198)
X^b^	Stop	(-1.5993)	(1.672)	(-1.5833)	(-2.6562)
Y	Tyr	5.4 (-0.8039)	-1.3 (0.2909)	136 (1.207)	5.66 (-0.2222)

^*a*^ Raw values were obtained from[47] Haig D, Hurst Land normally scaled indices are in parenthesis. Stop signal was excluded from normalization.

^*b*^ Stop signal, arbitrarily assigned values that are the least polar, most hydrophobic, largest in volume, and most negatively charged so that its distances from amino acids are greater than the distance between any two amino acids.

Second, the neural network was initialized with uniformly distributed random activities centered at 0.5 [44]. The network was then optimized by following the simulated annealing algorithm with preset values of *d*_*p*_ (e.g., *d*_*p*_ = 0.7) and *T* (e.g., *T* = 0.1), determined experimentally to maximize the proportion of valid tour paths (Eqs 2 & 3) [46]. Each resulting optimal tour path was checked for validity to ensure that all amino acids were covered and each amino acid was visited only once. Invalid paths were discarded and valid paths were saved for further analysis.

Third, we mapped the amino acids from an optimal path produced by the Hopfield network to the 64 possible codons. Because there are more codons than amino acids it was necessary to assign multiple codons to a single amino acid.

#### Codon wheel

We constructed a linear order of codons with minimal evolutionary distances by following known molecular evolutionary principles. First we gave preference to mutations at the 3^rd^ codon position and then to those at the 1^st^ codon position, followed by those at the 2^nd^ codon position, reflecting increasing evolutionary distances of nucleotide substitutions from the 3^rd^, to the 1^st^, and to the 2^nd^ codon positions. Second, for mutations introduced to the same codon positions, we gave preference to transitions over transversions, reflecting the fact that transitions occur more frequently than transversions [22]. Following these two rules, a linear sequence of codons, created by cycling the four bases at each codon position in the order of, e.g., “AGCTTCGA”, to minimize the total number of transversions, were uniquely defined and shown as a circular codon wheel (Fig 1). Note that the codon wheel could alternatively be defined by any of the other three possible transversion-minimizing base-cycling sequences: “AGTCCTGA”, “GACTTCAG”, or “GATCCTAG”. The resulting optimal codes would vary but be similar in having the shortest cumulative amino acid distances. Fig 1B shows one of such optimal codes.

To assign multiple codons to the same amino acid, we started with an arbitrary codon in a codon wheel (e.g., “AAA”) and traveled clockwise through all codons, while labeling each codon with an integer determined by the order of distinct amino acid to which this codon is assigned according to SGC (Fig 1). In other words, we assigned each codon an “SGC address”. For example, starting from “AAA” in a codon wheel shown in Fig 1, the codons were labeled as “AAA” (1), “AAG” (1), “AAC” (2), “AAT” (2), “GAT” (3), “GAC” (3), “GAG” (4), “GAA” (4), and so forth and ended with “ATT” (19), “ATC” (19), “ATG” (20), and “ATA” (21). This way, an optimal amino-acid path generated by a Hopfield network could be assigned to 64 codons based on tour positions. For example, if Lysine (K) has a tour position of 4 in a TSP path, it will be assigned to two codons “GAG” and “GAA”, both of which have an “SGC address” of 4 (although Lysine codons are “AAA” and “AAG” in SGC). Note that the SGC addresses of codons were arbitrarily assigned as long as they follow the order of a codon wheel. If tour positions of amino acids were random (i.e., not optimized by Hopfield network), this scheme is equivalent to a random permutation of amino acids among synonymous codon blocks [26]. A Hopfield-optimized tour path of amino acids, on the other hand, is expected to maximize the chance that similar amino acids are assigned to similar codons. In all cases, the choice of the initial codon determines the identity but not the error rate of the evolved codes.

### Statistical analysis of genetic codes

#### Randomized genetic codes

To test optimality of SGC and simulated genetic codes, we generated random codes as statistical controls by permuting the 20 amino acids and the stop signal among the 21 synonymous codon blocks [26]. This randomization scheme is a stringent test of code optimality. It maintains the same codon degeneracy as in SGC while removing any correlation that might exist between amino acids and codon blocks [26].

#### Code optimality measured by mutational error

**Fig 2:**
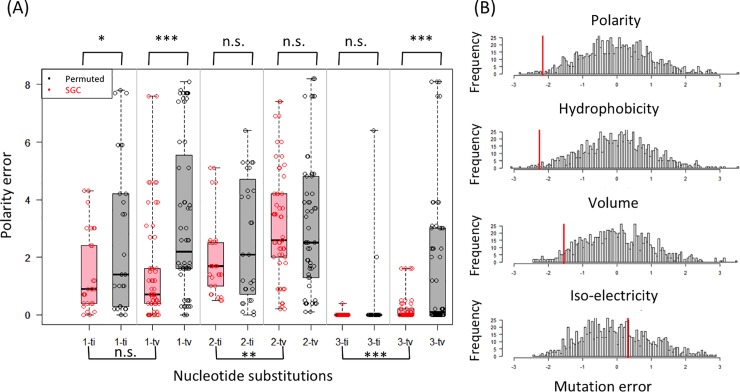
Patterns of mutation-error minimization in SGC. **(A)** On the x-axis, single-nucleotide mutations between pairs of codons are categorized by codon positions (1, 2, and 3) and by transitions (ti) or transversions (tv). The y-axis shows the errors caused by such mutations as quantified by the polarity index (Table 1). Points in red (n = 263) were derived from SGC; points in black (n = 258) by a single round of random permutation of amino acids among the synonymous codon blocks. **(B)** Distributions of amino-acid errors caused by single-nucleotide substitutions (calculated by Eq 4 with ti/tv = 5) from 1000 permuted codes. These error distributions show SGC significantly reduces mutation errors for polarity (p = 0.010, *t*-test), hydrophobicity (p = 0.011), and volume (p = 0.048), but not for isoelectric points (p = 0.648).

We quantified optimality of each code by calculating errors (i.e., changes in an index value) caused by single-nucleotide substitutions [47]. The mutational error of a code, a measure of overall code fitness, was the average error across all pairs of single-mutation codons [26]:

$$\Delta =\frac{\sum_{i=1}^{61}\sum_{j=1}^{9}w{|X}_{i}-X_{j}|}{n}$$ (4),

where *i* was the source codon, *j* was the destination codon (stop codons excluded) differing from the source codon by a single nucleotide, *w* was the transition/transversion ratio, which was set to 5 (Fig 2), *X*_*i*_ and *X*_*j*_ were the physio-chemical values of the two amino acids associated with the two codons, and *n* was the total number of one-nucleotide neighboring codon pairs. Statistical significance of the error of a code was assessed by the proportion of random codes with an equal or smaller error. Errors were also calculated individually at the three codon positions and for transitions and transversions separately as a way to estimate if errors were minimized at individual codon positions and for transition or transversion.

### Phylogenetic analysis

To explore biological basis of the self-learning algorithm, we inferred early evolutionary events during the origin of SGC using the tRNA sequences of *Pyrococcus furiosus* strain DSM_3638, a model hyperthermophilic archaebacterium. We downloaded the structurally aligned *P*. *furiosus* tRNA gene sequences from tRNAdb [48]. Redundant sequences were removed and an approximate maximum likelihood phylogenetic tree was obtained with FastTree using default settings [49]. Using the BpWrapper BIOTREE utility, branches with low bootstrap support (<0.7) were collapsed and the tree was rooted at the midpoint [50]. The phylogenetic tree was plotted using the APE package on the R/RStudio platform [51].We used phylogenetic autocorrelation to test co-diversification of tRNA sequences with their cognate amino acids. Phylogenetic autocorrelation is a measure of association of a variable with a phylogeny, in the same way as spatial autocorrelation being a measures of the influence of a variable by geographic distances [52,53]. We use the *gearymoran* function in the ADE4 package to calculate Moran's I with an amino acid index (e.g., polarity) and obtained its statistical significance with Geary's randomization protocol [54].

## Results

### Two sources of error minimization in SGC

There are a total of 263 pairs of amino-acid encoding codons that differ by a single base. These codon pairs could be categorized into six types according to the position of the differing base and whether it is a transition (A/G or C/T) or transversion (A/C, A/T, G/C, or G/T). Transitions occur more frequently than transversions, known as the transition/transversion mutational bias. When the error magnitude (measured by e.g., amino-acid polarity; Table 1) between two codons in SGC were plotted for each of the six categories, it was apparent that mutations at the 3^rd^ codon position caused the least errors, followed by the 1^st^ codon position and then by the 2^nd^ codon position (red boxes in Fig 2A). This was largely due to codon degeneracy by which multiple codons code for the same amino acid, which decreased in the order of the 3^rd^, 1^st^, and 2^nd^ codon positions and was over-represented by transitions. Codon degeneracy, however, is not itself adaptive because some form of degeneracy is inevitable for any genetic code consisting of more codons than amino acids.

If SGC does not minimize errors between neighboring codon blocks, one would expect the mutational errors similar between SGC and a randomized code. In reality, errors in SGC (measured by, e.g., errors in polarity) were significantly reduced relative to the permuted codes for transitions and transversions at the 1^st^ codon position (*p =* 2.9e-2 and 8.7e-5 by *t*-tests) and transversions at the 3^rd^ codon position (*p =* 1.4e-5), while there was no significant error reduction at the 2^nd^ codon position (Fig 2A). This pattern of error reduction indicates that SGC minimizes errors caused by mutations at the 1^st^ and 3^rd^ codon positions in addition to codon degeneracy at these positions. At the 2^nd^ codon position, although there was neither codon degeneracy nor significant error reduction relative to the random code, transitions caused significantly less errors than transversions (*p =* 1.3e-3).

In sum, two sources of error minimization in SGC were identified: (*i*) SGC significantly reduces mutation errors at the 1^st^ codon position and transversion errors at the 3^rd^ codon position and (*ii*) at the 2^nd^ and the 3^rd^ codon positions, errors by transitions are significantly minimized relative to those by transversions. These two rules were used to construct a codon wheel that minimizes evolutionary distances between codons (Fig 1).

### SGC co-minimizes errors in amino acid polarity, hydrophobicity, and volume

**Fig 3:**
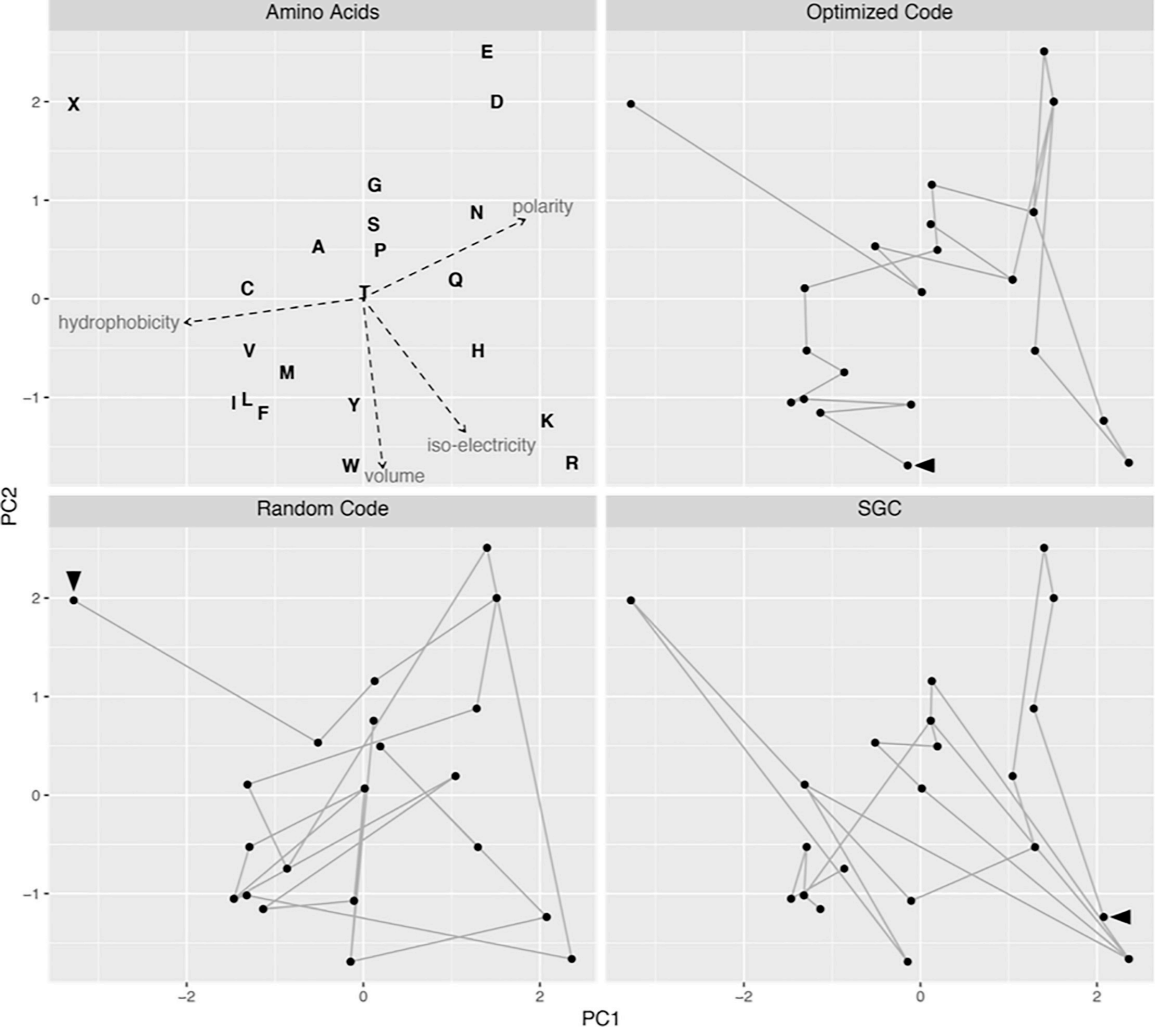
Hopfield network minimizes amino acid tour lengths. (**Top left**) First two principal components of the four amino acid indices (Table 1). The variances explained by the 1^st^ and 2^nd^ principal components are 48.73% and 37.96% respectively. (**Top right**) Amino acid path of a Hopfield-simulated code optimized for polarity and volume (tour lengths 32.2±4.4 in polarity error). (**Bottom left**) Amino acid path of a randomly permuted code (tour lengths 56.3±4.6, sample size 1000). (**Bottom right**) Amino acid path of SGC (tour length 41.0).

We tested the overall errors of SGC relative to random codes for the four major amino acid indices (Table 1). Results indicated that SGC significantly minimizes errors in polarity (*p =* 0.010, by *t*-test), hydrophobicity (*p =* 0.011), and volume (*p =* 0.048), but not iso-electricity (p = 0.648) (Fig 2B). The co-minimization of polarity and hydrophobicity was not surprising because these two indices were significantly anti-correlated with correlation coefficient -0.81, according to a principle component analysis of the four indices (Fig 3A).

### Hopfield-optimized genetic codes

**Fig 4:**
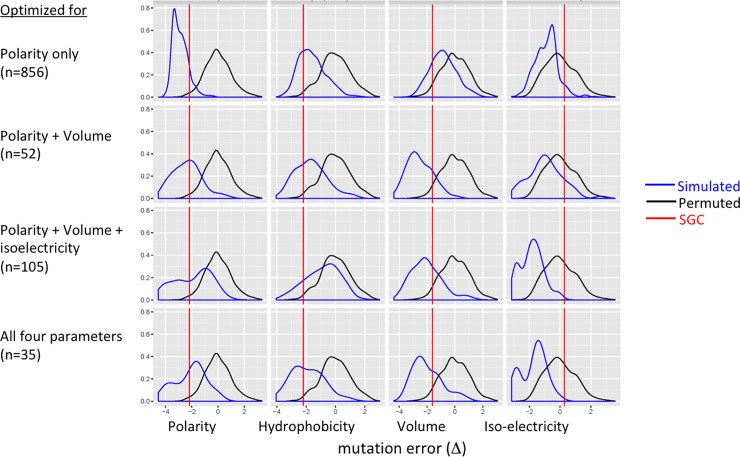
Hopfield network minimizes mutation errors. We constructed a 21-by-21 TSP and searched for the shortest paths traversing the 20 amino acids and the stop codon (21 “cities”) using Hopfield neural networks (see Methods). Distances between the “cities” were determined by either polarity alone (**top row**), or a combination of 2, 3, and all 4 parameters (**other rows**). Valid paths (number in parenthesis) were mapped to the codon wheel resulting in simulated genetic codes. Each simulated code was calculated for average single-mutation error rates based on each parameters (Eq 4). All error rates were normalized according to the mean and standard deviation of random codes (as in Fig 2B) and their distribution were shown as density plots. While it is possible to obtain codes optimized for all four parameters (**bottom row**), the most parsimonious codes with errors similar to SGC errors were generated with the Hopfield network that optimized for polarity alone (**top row**). An alternative way to visualize reduced errors in Hopfield-optimized codes is to show each code as an amino acid tour in a 2-dimensional principal component space, which shows significantly shorter tour lengths of Hopfield-optimized codes than those of permuted code and SGC (Fig 3).

There are 4 x 10^84^ possible genetic codes [55]. We used Hopfield networks to search for optimal genetic codes with the goal of assessing the possibility that SGC may have originated by a similar self-learning process. None of these codes was the SGC, but was comparable to the SGC in terms of its optimality. First, we obtained optimal codes by minimizing the total distance measured by a single parameter. For example, a total of 856 valid paths were generated from a run of 2500 rounds of simulations using amino acid distances determined by polarity. Indeed, the polarity errors of the simulated codes decreased significantly relative to the random codes and the mean error rate was close to that of SGC (-2.0 standard deviation from the mean error of random codes) (Fig 4A). The errors for hydrophobicity, volume, and iso-electricity of simulated codes were incidentally reduced. Adding volume as an extra distance parameter had similar error-reducing efforts to all four indices, while the decrease in volume was the largest (-3.0 standard deviation from the mean error) (Fig 4B). Continuing by adding iso-electricity as the 3^rd^ parameter, the simulated codes improved upon the random codes for all three measures of errors (Fig 4C). Finally, including all four parameters resulted in simulated codes optimized for all parameters (Fig 4D).

It could be concluded from these simulations that Hopfield network optimized genetic code in a highly sensitive manner depending on which and how many indices were included in the distance function. Further, considering the large iso-electricity error rate of SGC (0.3 standard deviation greater than the random error, Fig 2B), we rejected the hypothesis that SGC evolved by minimizing errors in isoelectric point. Indeed, it is most parsimonious to conclude that SGC evolved by minimizing the polarity error alone, with reduced errors in hydrophobicity and volume as incidental consequences (Fig 4A). This hypothesis was supported by the fact that optimization with respect to hydrophobicity or volume alone resulted in poorer match of errors between SGC and the simulated codes.

**Fig 5:**
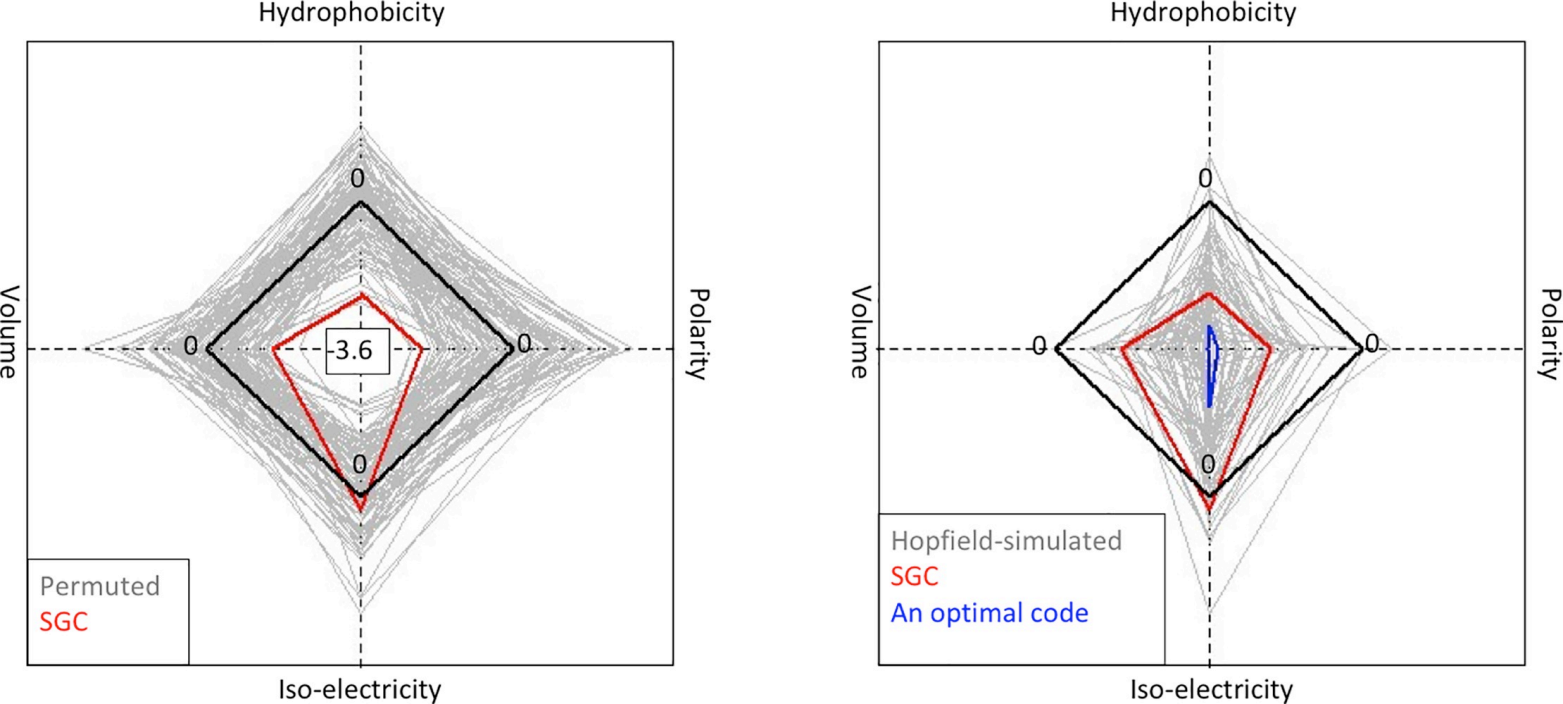
Hopfield network yielded genetic codes more optimal than SGC. (**Left Panel**) In this alternative display of error rates of genetic codes, each code is represented by a quadrilateral with vertices defined by the four errors. The black diamond represents the average errors of the permuted codes (n = 100). Note that the minimum error (-3.6 standard deviation from the mean) is placed at the center for all four parameters so that the smaller a quadrilateral condenses towards the center the more optimal a code is. (**Right Panel**) Gray quadrilaterals represent Hopfield-simulated codes (n = 52) optimized for polarity and volume (the same output used for Fig 4, 2^nd^ row). The simulated codes have generally less errors than randomized codes. Many are more optimal than SGC, one of which is highlighted in blue and its coding pattern is shown with a codon wheel (Fig 1B).

Next, we searched for simulated codes that were more optimal than SGC by plotting the error rates grouped by individual codes (Fig 5). One such code with all four errors less than -2.0 standard deviation away from the mean random errors was identified and its codon assignment was visualized with a codon wheel (Fig 1B). This Hopfield-optimized code was more optimal than SGC in that, e.g., all non-polar amino acids were mapped to codons with the 2^nd^ codon position being a purine (A or G). Furthermore, all positively or negatively charged amino acids were mapped to codons with the 2^nd^ codon position being a thymine (T). Most significantly, genetic codes similarly or more optimal than SGC were not a rarity but emerged readily from a Hopfield network (Fig 5), suggesting that SGC was suboptimal in error minimization.

### Phylogenetic autocorrelations

So far we have shown that SGC was an unsurprising (and indeed suboptimal) outcome of a Hebbian learning process when operating under the same set of biological constraints as in SGC. Here we explored the possible biological basis of Hebbian learning during the origin of SGC by examining footprints of early evolutionary events left in the gene sequences of the contemporary protein-translation system from the archaebacterium *Pyrococcus furiosus*.

**Fig 6:**
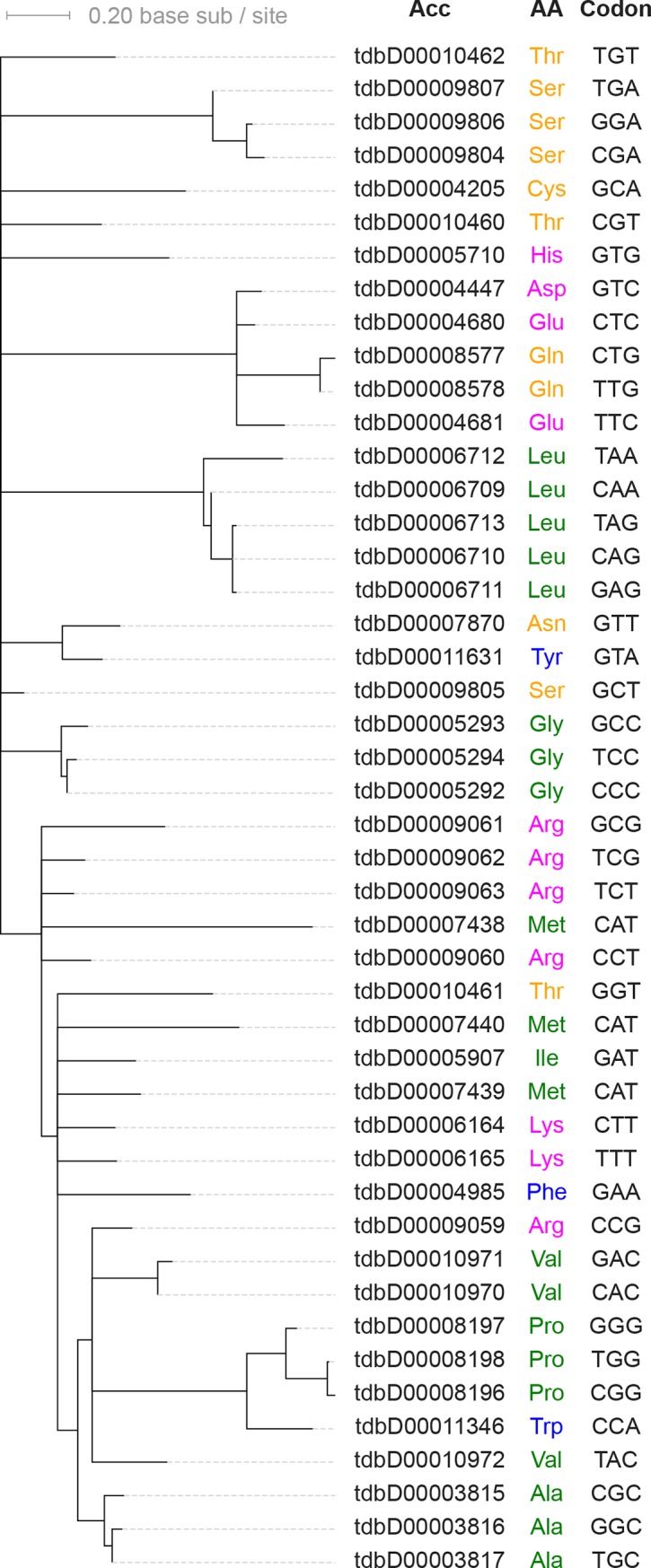
Molecular phylogeny of *Pyrococcus furiosus* tRNAs. Leaf nodes are labeled with the cognate amino acid names and the anticodon sequences. Amino acid names are colored according to an amino acid physio-chemical classification as in Fig 1 [18]. Only significantly supported branches (bootstrap value > = 0.7) are shown (see Methods). Phylogenetic autocorrelation with amino acid indices are all significant (Moran’s I = 0.1973 with *p* = 8.1e-7 for polarity, I = 0.21517 with p = 1.6e-07 for hydrophobicity, I = 0.1593 with p = 6.0e-05 for volume, I = 0.1116 with p = 2.4e-3 for isoelectricity), suggesting early co-diversification between tRNAs and physio-chemistry of cognate amino acids.

Recognition of amino acids by codons–is mediated by tRNAs, each of which is ligated by an aminoacyl-tRNA synthase (aaRS) at the acceptor stem to a specific amino acid according to the anticodon sequence [56]. Unlike the aaRSs, tRNAs are structurally homologous, suggesting a single common origin. Phylogenies of contemporary tRNA gene sequences show a general monophyly of iso-accepting tRNAs (i.e., tRNAs recognizing the same amino acids) although codon recruitments from different tRNA clades have occurred (Fig 6) [57]. Further, significant phylogenetic autocorrelation of tRNA with the physicochemical properties of cognate amino acids supports that expansion of iso-accepting tRNA groups to all twenty amino acids involved similar tRNAs recognizing physio-chemically similar amino acids (Fig 6). This autocorrelation is consistent with an early co-diversification for tRNA sequences and cognate amino acids.

## Discussion

This study was motivated by the proposition of evolutionary connectionism that algorithmic learning could lead to self-optimized, adaptive, and robust living systems [10,11]. Using the origin of genetic code as a test case, we show that it is indeed plausible for an error-minimizing genetic code to emerge through a Hebbian learning process without natural selection playing a role at system levels.

### TSP representations of genetic code

Our algorithm for finding optimal genetic codes consisted of two main steps. In the first step, we formulated code optimization as a combinatory optimization problem of finding the shortest paths traversing the 20 amino acids and the stop signal. This Traveling Salesman Problem (TSP) formulation of the genetic code naturally lend itself to be solved with the Hopfield neural network, which is an implementation of the Hebbian learning rule [43,44]. In the second step, we mapped the optimal tour positions emerged from the Hopfield network to a circular sequence of codon–a codon wheel (Fig 1)–that are based on biological constraints present in SGC (e.g., the codon degeneracy decreasing in the order of 3^rd^, 1^st^, and 2^nd^ codon positions and the size distribution of synonymous codon blocks). Note that there was no guarantee that optimized genetic codes emerged from the Hopfield network would be as optimal as SGC. This could be seen in randomly permuted codes, most of which showed higher error rates than SGC although generated with the same set of SGC constraints including codon-degeneracy, transition/transversion bias, and the size distribution of synonymous codons (Fig 2). More tellingly, plenty of codes optimized by the Hopfield network and mapped to the same codon wheel showed higher error rates than SGC (Fig 4).

Also using simulated annealing, DiGiulio *et al* [58] was able to achieve a code with an optimality ratio of 51.7% with respect to polarity, whereas we found a code with an optimality ratio of 48% with respect to polarity. Here we use DiGiulio’s optimality ratio: {*Δ*(*Mean*)−*Δ*(*SGC*)}/{*Δ*(*Mean*)−*Δ*(*Code*)}×100, where Δ(Mean) is the average error in polarity associated with a set of random code, Δ(SGC) is the error in polarity in the SGC, and Δ(Code) is the error in polarity the given code. DiGiulio *et al* used simulated annealing to minimize error directly without a neural network. On average 89.2% of our codes optimized for polarity were better than SGC with respect to polarity. The method however does not always produce optimal codes, as the simulated annealing method to solve the traveling salesman problem only finds local minima, which sometimes improve on SGC and sometimes do not.

Together, these two algorithmic steps (i.e., the use of a Hopfield network and a codon wheel) allowed us to establish that genetic code as optimal as SGC emerge quickly and without natural selection. It is conceivable that these two steps be combined into a single TSP algorithm. We have represented the genetic code as a 64 amino acids (with repetition)-by-64 codons TSP and set the distances between the same amino acids as zero. In practice, however, optimal codes emerged from such a Hopfield network turned out to be not strictly comparable to SGC (and its random permutations) because the size distribution of synonymous codon blocks was not preserved. One way of preserving the codon block size distribution in SGC is to number the codon blocks sequentially according to a codon wheel (Fig 1) so that a 21 amino acid– 21 codon block TSP could be constructed. This representation would be equivalent to the two-step algorithm we used because the “SGC address” of a codon was precisely the position of the codon block containing this codon in a codon wheel.

#### Coevolving molecular network resembles a Hopfield network

TSP is perhaps the most studied combinatorial optimization problem for which Hopfield network is one of numerous heuristic searching algorithms [45,59]. Our choice of Hopfield network is motivated by its embodiment of the Hebbian learning rule, a major self-organizing principle in evolutionary learning [15]. Similar to Hopfield network being a computational implementation of the Hebbian rule (Eq 1), the molecular machinery associated with the genetic code may well be a biological analog of the Hopfield network. Mirroring a Hopfield neuron associating a city with a specific tour position, each macromolecule (e.g., an tRNA) associates a codon with a amino acid. Further resembling Hopfield neurons inter-connected with varying degrees of weights, members of a macromolecule gene family (e.g., tRNAs and aaRSs) are related to each other with varying degrees of phylogenetic distances (Fig 6). Quantitatively, the rate of increase in the weight (*w*_*ij*_) of connection between two Hopfield neurons (*i* and *j*) with correlated (*r*) activities *x*_*i*_ and *x*_*j*_ could be expressed as: *dw*_*ij*_/*dt*~*r*(*x*_*i*_,*x*_*j*_) [43]. Similarly, a history of co-diversification among tRNAs and amino acids creates phylogenetic auto-correlation between gene sequences and amino acid physio-chemistry (Fig 6). The phylogenetic autocorrelation could be expressed as: *dL*_*ij*_/*dt*~*d*(*x*_*i*_,*x*_*j*_), where *L*_*ij*_ is the phylogenetic distance (i.e., tree length) between two paralogs and *d*(*x*_*i*_, *x*_*j*_) is the codon or amino acid distance associated with the pair of paralogs. Our Hopfield and Hebbian interpretations of the origin of SGC are consistent with the codon capture hypothesis, which posits that primordial codon expansion followed a phylogenetic path of minimum changes in amino acid physio-chemistry or exchangeability [60–62]. Indeed, before developing his namesake network, Hopfield himself had proposed an origin of SGC through co-diversification of tRNA molecules and their binding specificities [63].

To summarize, the phylogenies of macromolecule gene families resemble Hopfield networks and may thus be considered a recapitulation of the self-optimizing process of the SGC.

### Self-learning in evolution of multi-gene families

Molecular systems consisting of co-diversifying paralogs are not limited to the genetic code. In fact, genome and gene duplications coupled with neo-functionalization or sub-functionalization are a pervasive and predominant mechanism of evolutionary innovation [64–66]. For example, duplications of the and the α- and β-hemoglobin genes have led to novel capacities for binding oxygen and carbon dioxide [65]. Duplication and sub-functionalization of the homeobox genes contributed to body plan diversification in bilaterian animals [67]. Vertebrate olfactory sensing system consists of rapidly evolving members of the odorant receptors gene family [68]. Hopfield himself proposed a neural computational model akin to his namesake network that distributes odor recognition among a large number of sensory cells [69]. The Major Histocompatibility Complex (MHC) loci responsible for adaptive immunity in vertebrates consists of multi-gene families [70]. For the parasites, genomes of microbial pathogens such as *Trypanosoma* and *Borrelia* are enriched with duplicated surface antigen genes for defense against host immunity [71,72].

In each of these cases, a complex adaptive molecular system has evolved from phylogenetic co-diversification between genes and gene functions. The present work shows that Hopfield network offers a way to simulate and perhaps to design artificial self-optimizing genetic systems.
